# Multifocal Buruli Ulcer Associated with Secondary Infection in HIV Positive Patient

**DOI:** 10.1155/2013/348628

**Published:** 2013-12-25

**Authors:** Kassi Komenan, Ecra J. Elidjé, Gbery P. Ildevert, Kouassi I. Yao, Kouame Kanga, Kouassi A. Kouamé, Sangaré Abdoulaye, Kourouma S. Hamdam, Yoboué P. Yao, Kanga Jean-Marie

**Affiliations:** Department of Dermatology and Infectiology, School of Medicine, Felix Houphouét Boigny University of Abidjan, BP 01 V166 Abidjan 01, Cote d'Ivoire

## Abstract

Buruli ulcer is a chronic and infectious skin disease, caused by *Mycobacterium ulcerans*. It leads to large skin ulceration and sometimes bone infection which is responsible for deformities. Here, we report a case of multifocal form of Buruli ulcer associated with secondary infection in a 46-year-old human immunodeficiency virus (HIV) positive woman. The antimycobacterial drugs combined to surgery allowed curing this multifocal case and rose up two relevant issues: the susceptibility of immune reconstitution inflammatory syndrome (IRIS) occurrence and *Mycobacterium* dissemination. The deep immune depression, the underline biological, and clinical disorders of the patient might contribute to IRIS occurrence and Buruli ulcer dissemination. Future investigations have to be conducted on the mechanism of IRIS on set and on *Mycobacterium ulcerans* dissemination after ARV drugs initiation and the patient related underline clinical or biological disorders.

## 1. Introduction

Buruli ulcer is mycobacterial disease, caused by *Mycobacterium ulcerans*. It is an emerging and neglected tropical disease marked by devastating skin ulceration, and sometimes by bone lesion. Buruli ulcer represents a public health problem in Cote d'Ivoire, where it remains endemic. Until recently, surgery was the only treatment for this disease [1].

Since 2004, significant progress has been made and Buruli ulcer is treated by rifampicin and streptomycin [2]. Multifocal forms of BU are rarely described in the literature and they are sometimes associated with secondary infection and human immunodeficiency virus (HIV) coinfection [3]. When associated with HIV coinfection, the use of antiretroviral treatment (ART) has led to substantial decrease in HIV-related morbidity and mortality, through preserving the host's immune system and reducing opportunistic infections. However, complications associated with ART initiation, including immune reconstitution inflammatory syndrome (IRIS), are also increasingly described [4, 5]. Sometime, this condition worsens ART initiation, mainly in developing country such as Africa where ART is now available [6].

The disseminated process of *Mycobacterium* is not fully understood, even if it was reported that it may be caused by IRIS [7].

Here, we report a case in adult woman, who was hospitalized in the Department of Dermatology at the Teaching Hospital of Treichville, for a multifocal form of BU associated with secondary infection and HIV coinfection.

## 2. Case Report

It was a 46-year-old female patient, living in an endemic area of BU (western part of Côte d'Ivoire) who was hospitalized for several ulcerations on the two legs.

According to her, the disease started by nodules on the left leg, associated with pain, hot edema, and local redness. Two weeks later, the lesions evolved into a large ulceration. As for this ulceration, she used some traditional medicines before going to the community base medical center where an antibiotic (none precised) was given to her and the wound was dressed, without success.

Later, she observed new ulcerations on the right leg, and, on the left one, the lesions extended and become painful with fever. Then, the patient decided to go to the Dermatology Department for better management.

The clinical examination was as follows.

**Figure 1:**
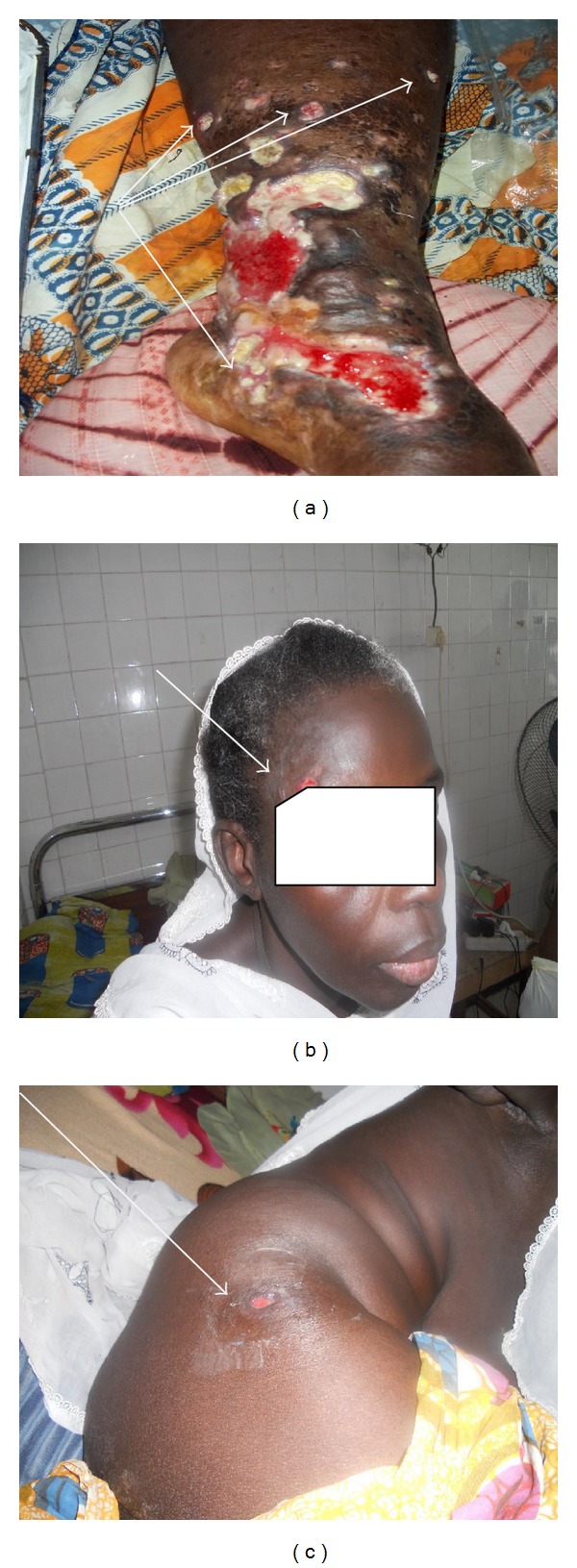
(a) A large ulceration of the left leg associated with pain, redness, and hot edema and several small ulcerations with identical features to the large one. (b) and (c) Several painless ulcerated nodules appeared on the body (head and bottom) after antiretroviral treatment initiation.

- The general state of our patient was distorted with a blood pressure of 130/80 mm Hg, a pulse of 88 beat/mn, and a temperature of 38.7°C, associated with minor cutaneous dehydration. Her weight was 63 kilograms.
- Local examination showed the following.
  - On the left lower limb, there is a large ulceration (10 cm of diameter) that extends to the left internal malleolus. The ulceration depth was dirty, suppurative, yellow, and necrotic. Black necrotic eschar with irregular border in different areas of the ulcerations was associated with pigmented surrounding skin. This ulceration was associated with pain redness and hot edema extended to the foot. Several small ulcerations with identical features to the large one were observed (Figure 1(a)).
  - On the right leg, we noted a large ulceration (12 cm of diameter) with identical features to the left one.
  - No lymph node and lymphangitis were found. The peripheral pulse and sensibility of the two lower limbs were normal.

We suspected secondary wound infection (bacterial) based on the following features: a general distorted state, an ulcerated, suppurative, and necrotic wound with edema, local redness, heat, pain, and fever.

We performed blood check examinations to confirm the diagnosis, which showed a leukocyte count of 11000 cells/mm^3^, an anemia with 6.3 g/dL, a biological inflammatory syndrome, and hypoprotidemia. Kidney and liver biological examination and blood sugar level were normal. The X-ray of the lower limbs did not find any bone lesion.

Patient was taken to surgical emergency room to perform surgery.

In a local level, a surgical excision and debridement of necrotic tissues were performed on the two legs. Sample and pus were taken from the necrotic tissues for microbiological examination including polymerase chain reaction (PCR). The wound was dressed by antiseptic.

In the systemic level, the patient was put under clavulanic acid + amoxicillin, 2 grams (g) per day, added to Paracetamol of 1 g three times per day for 2 days, blood transfusion of 450 milliliters (mL) one time, and intravenous rehydration of 2 liters per day during 2 days. Ferrous was given on the third day: 20 milligrams (mg) orally per day.

After 48 hours, the bacteriological examination of the pus taken from the wound showed some streptococcus, the polymerase chain reaction (PCR) examination of the biopsy stain showed *Mycobacterium ulcerans*, and histopathological slides showed inflammatory cell with acid fast bacilli.

In addition, the HIV test was performed after counseling and the patient consent was positive for HIV-1 with CD_4_ cells count of 51 cells/mm^3^.

We concluded to multifocal Buruli ulcer form associated with secondary wound infection to streptococcus in HIV-1 positive patient.

The patient was treated by streptomycin (80 mg intramuscular, one daily injection) combined to rifampicin (600 mg orally, once per day).

For the HIV-1 infection, the patient received ART by combination of Stavudine (40 mg) + Lamivudine (150 mg) + Nevirapine (200 mg) twice a day during 2 weeks. For its good tolerance and after liver and blood kidney test, this combination was pursued.

One month later, several painless ulcerated nodules appeared on the body (Figures 1(b) and 1(c)).

The PCR and histological examinations confirmed *Mycobacterium ulcerans* infection.

The CD_4_ cell count that moment was stable, 51 cells/mm^3^.

We hypothesized that it was either an immune reconstitution inflammatory syndrome due to *Mycobacterium ulcerans* or an inefficient ARV.

We decided to change the ARV combination; we used Truvada (Emtricitabine 200 mg + Tenofovir 245 mg) once a day combined to Kaletra (Lopinavir 200 mg + Ritonavir 50 mg) twice a day orally. All the ulcerated nodules were excised and the antimycobacterial treatment was pursued over the 8 weeks as recommended by WHO.

Two months later (from the beginning of the ART), half of the lesions were cured; the CD_4_ cell count gain was not significant (53 cells/mm^3^). Unfortunately, we were not able to perform viral load (which allow for real evaluation of the ARV efficacy), and the hemoglobin was 9.8 g/dL; the patient general state was good with neither fever nor cutaneous dehydration.

**Figure 2:**
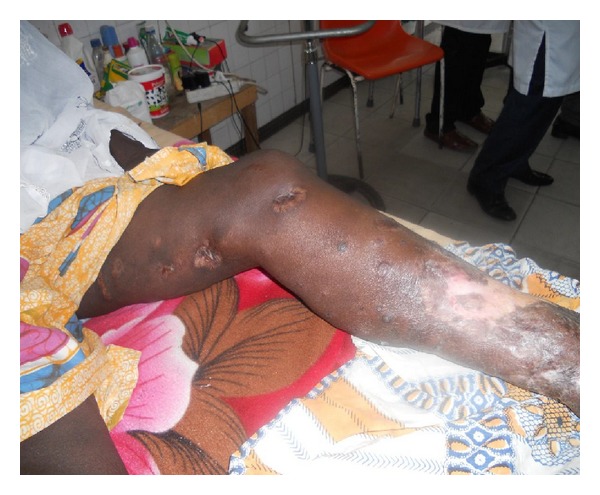
Healed multifocal form of Buruli ulcer (left lower limb) at 3 months of followup.

At 3 months (from the beginning of the ART), CD4 cell count increased to 82 cells/mm^3^. Blood check found hemoglobin to be 10.9 g/dL, and liver and kidney functions were normal. We observed a total cicatrization of Buruli ulcer lesions (Figure 2) and stopped the antimycobacterial treatment.

At 6-month follow-up time, CD_4_ cell count was 120 cells/mm^3^ and biological ART followup did not found any abnormality.

## 3. Discussion

The two relevant aspects of this case report are, firstly, the mechanism of *Mycobacterium ulcerans* dissemination in this multifocal form of BU and, secondly, the difficulty to conduct concomitantly BU treatment and ART.

The use of ART has considerably reduced HIV related mortality, in terms of both opportunistic disease reduction and preservation of patient immune system [6].

However, quantitative and qualitative recoveries of pathogen-specific cellular and humoral responses have been noted to multiple opportunistic pathogens including *Mycobacterium* (*tuberculosis*, *leprea*, *avium*, etc.) [8].

This paradoxical reaction or Immune reconstitution inflammatory syndrome (IRIS) is recognized as a potential complication that has been previously observed in HIV-positive patient associated with *Mycobacterium* infection. The frequency of its occurrence is 10 to 25% of patient is that received ART [8]. In Africa, this phenomenon became more and more observed, due to ARV availability.

The interval between ART initiation and IRIS occurrence is highly variable, from one to several weeks. But, the majority of IRIS occurs in patient with low mean CD_4_ cell counts (under 50 cells/mm^3^). Its onset is marked by paradoxal worsening of clinical or laboratory parameters despite favorable development of the surrogate markers [6]. The reaction may arise in 2 different settings, depending on whether ART was started in a patient treated for ongoing opportunistic infections or in a clinically stable patient [8, 9].

Few cases of disseminated BU forms associated to HIV coinfection were published [3, 10].

In our case, we started BU treatment two weeks before initiating ART, and the paradoxal reaction or IRIS appeared one month later, associated with fever and swelling.

The CD_4_ cell count at the time of ART initiation was low (51 cells/mm^3^) and the patient general state was distorted associated to biological disorders.

This alliterated condition of the patient might favor the onset of the paradoxal immune reaction. It may explain the emergence of disseminated nodules of BU after ART initiation.

At one month of ART, The none significant CD_4_ cells count (53 cells/mm^3^) and the several BU and ulcerated nodules allowed hypothesizing ART failing with *Mycobacterium* dissemination favored by IRIS onset. Unfortunately, viral load was not available in our setting, but we did not discard these hypotheses.

So, we retained ART failing under Stavudine (40 mg) + Lamivudine (150 mg) + Nevirapine (200 mg) even if we could not perform viral load in our setting. Therefore, the patient was treated by another first line ARV combination using Truvada (Emtricitabine 200 mg + Tenofovir 245 mg) associated with Kaletra (Lopinavir 200 mg + Ritonavir 50 mg) and added 4 weeks of antimycobacterial drugs treatment because of disseminated lesions (12 weeks duration of treatment). Under this treatment, the CD4 cell counts increased progressively at 2 months, at 3 months, and at 6 months (53 cells/mm^3^, 83 cells/mm^3^, and 120 cells/mm^3^).

In addition, all Buruli ulcer lesions were cured; the patient general state was significantly improved, including the anemia, the dehydration, and the hypoprotidemia.

So, the underline conditions of the patient might contribute to the onset of IRIS and the *Mycobacterium* dissemination in this HIV-1 coinfection case. The distorted general state of the patient added to the traditional medicine applied on the wound contributed to the secondary wound infection occurrence.

Moreover, ARV combination used in this case (Stavudine (40 mg) + Lamivudine (150 mg) + Nevirapine (200 mg)) did not work because of Rifampicin usage. This drug decreases nevirapine Cmax and Cmin. That must have explained why this ARV combination failed in our case [11].

## 4. Conclusion

This case report highlights the difficulty to treat multifocal form or Buruli ulcer associated with secondary wound infection and HIV coinfection. This difficulty is related to possible occurring of immune reconstitution inflammatory syndrome and *Mycobacterium ulceran* dissemination which needs to be well understood. Therefore, future studies need to be conducted both on the mechanism of IRIS onset and on *Mycobacterium ulcerans* dissemination in order to improve the management of HIV infected patients.
